# IL-39 and IL-35 gingival crevicular fluid levels in diabetic patients with generalized periodontitis

**DOI:** 10.1007/s00784-023-05484-3

**Published:** 2024-01-30

**Authors:** Sandy shabaan Hassan, Maha Abdelkawy, Olfat Gamil Shaker, Nayroz Abdel Fattah Tarrad

**Affiliations:** 1 Oral Medicine and Periodontology Department, Faculty of Dentistry, Fayoum University, Fayoum, Egypt; 2 Ahram Canadian University, 6th of October City, Egypt; 3 Oral Medicine and Periodontology Department, Faculty of Dentistry, Beni-Suef University, Beni-Suef, Egypt; 4 Medical Biochemistry and Molecular Biology Department, Faculty of Medicine, Cairo University, Giza, Egypt

**Keywords:** Periodontitis, Diabetes mellitus, IL-35.IL-39, Non-surgical periodontal therapy

## Abstract

**Objectives:**

This research was performed to investigate if there is a role for IL-39 in immunopathogenesis of both systemically healthy and diabetic periodontitis patients. Additionally, to explore if we can consider IL-39 and IL-35 as biomarkers for periodontitis activity.

**Materials and methods:**

A total of 38 periodontitis patients and 19 control volunteers were included in our study. The periodontitis patients were divided equally into (Group I), 19 patients with stage III grade C periodontitis with diabetes mellitus and (Group II), 19 patients with stage III grade B periodontitis and systemically healthy. Gingival crevicular fluid levels of each interleukin were measured pre- and postoperatively for all periodontitis patients as well as control subjects using ELISA.

**Results:**

Our study results showed that the highest level for IL-39 was in diabetic periodontitis patients that decreased significantly postoperatively. However, the highest level for IL-35 was revealed in control group while the lowest value was registered in diabetic periodontitis patients and statistically increased after periodontal treatment.

**Conclusions:**

Based on the results of our research, both investigated biomarkers may have a potent role in pathogenesis of periodontitis.

**Clinical relevance:**

We could consider both interleukins as accurate diagnostic markers for periodontitis patients, regardless of diabetes mellitus association, as well as promising markers that can aid in the prevention and treatment of periodontitis patients worldwide.

## Introduction

Periodontitis is the sixth most prevalent disease and is a form of bone pathology worldwide. Periodontopathogens in dental plaque are the leading cause of the disease. The destructive procedures of periodontitis develop out of interplay between the dysbiotic community and imbalanced host immune response, in addition to genetic, systemic, and environmental modifying risk factors leading to destruction of periodontal tissues, in addition to acting as a tempering factor of the systemic health of the patient [1, 2].

Periodontitis is associated with an array of systemic diseases and conditions such as diabetes mellitus (DM). Both are chronic diseases, with common etiopathogenic and pathophysiological associations. The relationship between periodontal disease and DM is bidirectional. Periodontal disease is currently considered the sixth complication of DM, and periodontitis affects the prevalence, progression, and therapeutic management of DM. In addition, inflammation is an essential factor in both periodontitis and DM, and its contribution to the development of both diseases is well-recognized [3, 4]. Also, it has been clearly established that those cytokines produced by immune-inflammatory cells, either pro-inflammatory or anti-inflammatory play a significant role in the pathogenesis of periodontal disease, thus driving destruction, remodeling, progression, and repair of the periodontal apparatus [5].

IL-35 is a member of the IL-12 cytokine family and a novel immune-suppressing cytokine mainly produced by regulatory T cells (Tregs). It is formed from a heterodimer of IL-12p35 subunits and Epstein-Barr virus induced gene 3 (EBI3). The known functions of IL-35 include the suppression of T cell proliferation by blocking mitosis in the G1 phase without eliciting apoptosis and inducing the development of IL-35-producing T cells (iTr35) which are subsets of regulatory T cells. In addition, it hampers the differentiation of Th17 cells and inhibits IL-17 synthesis. Thus, it is closely associated with various immunological and infectious diseases such as rheumatoid arthritis, asthma, and inflammatory bowel disease [6–8].

IL-39 is the most recently discovered IL-12 family member and is a 54-kDa heterodimer composed of IL-23p19 and Ebi3 subunits. As mentioned in the literature, it is secreted by B cells that have been stimulated by lipopolysaccharide, with a positive correlation with the duration of stimulation, and its mRNA is expressed by dendritic cells and macrophages. Recent studies have revealed its proinflammatory role as it activates STAT1/STAT3 signal molecules by combining with the IL-23R/gp130 receptor to mediate inflammatory responses. Additionally, intercellular IL-23p19 enhances the surface expression of intercellular adhesion molecule-1 and vascular cell adhesion molecule-1 in endothelial cells, which augments the attachment of leukocytes and improves their transendothelial migration [9–11].

IL-39 has been studied in several diseases, such as systemic lupus erythematosus; it promotes a proinflammatory response that demonstrates its immunopathogenic effect [12]. Another study revealed an increase in number of B cells involved in production of IL-39 in mice with lupus suggesting that IL-39 induces differentiation and/or expansion of neutrophils in lupus-prone mice [13]. Moreover, results suggest that IL-39 promotes the growth of pancreatic cancer by promoting the growth and inhibiting apoptosis of cancer cells [14]. A recent study investigated the effects of IL-39 in a mouse concanavalin A-induced liver injury model and found that IL-39 amplified serum alanine aminotransferase and aspartate aminotransferase levels, inflammatory infiltration, and hepatocyte necrosis. Additionally, IL39 elevated the serum concentrations of interferon-γ, tumor necrosis factor-α, and IL-17α, eliciting a pro-inflammatory state [15].

Cumulative evidence supports that non-surgical periodontal therapy (NSPT) diminishes the prospect of inducing bacteremia caused by periodontitis and has a positive effect on systemic inflammatory status and metabolic control. Various interventional studies have demonstrated the beneficial effect of NSPT on glycemic control, as indicated by reduced glycosylated hemoglobin (HbA1c) and fasting blood glucose levels [16, 17]. In addition, a bacteriological result emphasized its role, where improvement was seen in all periodontal and metabolic parameters in the treatment group after NSPT [18]. The latest study aimed to evaluate the effects of NSPT on metabolic control, systemic inflammation, and cytokines in patients with T2DM with Stage III periodontitis, and concluded that successful NSPT tends to reduce the inflammatory burden within the local and systemic tissues via reduction of TNF-α, hs-CRP, blood glucose, and HbA1c, but increases the anti-inflammatory cytokine IL-10 [19].

The Gingival crevicular fluid (GCF) sample is characterized by its site-specific nature, efficiency, simplicity, and low cost. On the horizon, we could consider it a peridiagnostic tool, as its constituents might be correlated with clinical assessment, thus offering a basis for patient-specific diagnostic tests for periodontal disease. Interestingly, GCF, as a diagnostic modality, could indicate the presence of a disease process before extensive clinical damage occurs [20, 21].

Periodontitis and DM are common diseases in modern societies that have negative effects on health and quality of life. As evident in recent research, both are highly prevalent and expected to increase. Thus, seeking newer biomarkers that could help to establish the most appropriate prevention and treatment modalities is the challenge of research nowadays [4]. To the best of our knowledge, no data elucidate the relationship between periodontitis, DM, and IL-39; therefore, we intended to estimate its level to obtain an insight into the expected role of IL-39 in the immunopathogenesis of periodontitis patients with and without DM, as well as to estimate the level of IL-39 and IL-35 in our participants to evaluate the effect of  (NSPT) on the way to explore the role of these controversial cytokines, which are members of the same interleukin family, as biomarkers for periodontitis. Our hypothesis was that the GCF levels of the two estimated markers might show variations in the studied groups.

## Materials and methods

This study was conducted over a period of 6 months (November 2022 to April 2023). The participants were selected from the outpatient clinics of Ahram Canadian, Fayoum, and Beni Suef. The study procedures included clinical examination, sampling collection, treatment plan, and follow-up visits, which were explained to the enrolled patients who provided informed consent following the Declaration of Helsinki. This study was approved by the Research Ethics Committee of the Faculty of Dentistry, Beni-Suef University (approval number: REC-FDBSU/06102022–02/HS). In addition, the clinical trial was registered in the U.S. National Institutes of Health Clinical Trials Registry (ClinicalTrials.gov: NCT05880654).

### Study population

A total of 38 patients and 19 control volunteers were enrolled in the study. Patients with periodontitis were diagnosed based on the radiographic and clinical diagnostic criteria proposed by The 2017 World Workshop of the Classification of Periodontal and Peri-Implant Diseases and Conditions [22, 23]. Patients with diabetes were enrolled in this study according to the criteria of the American Diabetes Association [24]. Patients treated with any type of medication and/or antibiotics during the past 3 months, pregnant or lactating, and receiving professional periodontal treatment during the past 6 months. Current or former smokers (participants who quit smoking) [25] were considered among the exclusion criteria of our study subjects.

Our enrolled subjects were sorted into three groups as follow:

- Group I (Periodontitis with T2DM): Nineteen diabetic patients with more than 30% of the examined sites showed a clinical attachment level (CAL) ≥ 5 mm and were diagnosed with stage III grade C generalized periodontitis.
- Group II (Periodontitis Group): Nineteen systemically healthy subjects with more than 30% of the examined sites showed CAL ≥ 5 mm and were diagnosed with stage III grade B generalized periodontitis.
- Group III (control group): Nineteen systemically and periodontally healthy volunteers who presented to the restorative dental clinic with clinically healthy gingiva that showed nearly zero plaque index (PI), gingival index (GI), CAL, and ≤ 3 mm pocket probing depth (PPD) were enrolled.

### Periodontal examination

At the first visit, a complete periodontal clinical examination including PI, GI, PPD, and CAL was performed by a single calibrated examiner (S.H). GI and PI were recorded at four sites around the tooth (buccal, lingual/palatal, mesial, and distal), whereas PPD and CAL were measured at six sites on each tooth (mesial, mid, distal aspect of the buccal, and palatal/lingual sites). PI was established according to the description of Silness & Löe [26], and marginal gingival bleeding was registered as GI [27]. PPD was measured from the free gingival margin to the base of the periodontal pocket [28], CAL is the distance from the cementoenamel junction to the base of the periodontal pocket [29]. The obtained readings were rounded to the highest whole millimeter using a manual periodontal probe, the Michigan 0 probe with Williams’ markings.

### Periodontal therapy

Full-mouth supra-and subgingival scaling and root planning in two sessions were performed for all patients with periodontitis at two appointments on sequential days by same operator (SH), supragingival scaling using an ultrasonic device, and subgingival debridement under local anesthesia if needed using periodontal Gracey curettes (Lustra Gracey periodontal curettes, Dentsply, Surrey, UK). The motivation for self-performed plaque control measures using soft toothbrushes and interdental cleansing devices was clarified to all patients after treatment completion. Reassessment after 1 month for all patients with periodontitis, pre-operative sample sites that showed improvement in clinical parameters, PI, GI, and PPD, will be enrolled in our post operative GCF samples.

### GCF samples collection

Preoperative GCF samples were collected from the deepest pockets of each patient. Samples were assembled from patients before performing non-surgical periodontal therapy to avoid dilution of samples. GCF collection was performed using micropipettes, as this technique yields undiluted native GCF [21]. Micropipettes allow precise collection of GCF from a given site in a fixed volume [30] where the concentration of biomarkers can be estimated. The sampling area was isolated using cotton rolls to avoid contamination with saliva, and supragingival plaque was removed using a manual scaler, washed with a water spray, and dried. The micropipettes were inserted until mild resistance was observed. Saliva or blood-contaminated samples were excluded from the study. Similarly, postoperative samples were collected after 1 month. The samples were stored at − 80° Celsius until all study samples were collected for analysis.

### Determination of IL-35 and IL-39 levels in GCF

#### IL-35

The GCF level of IL-35 was measured using a Human Interleukin-35 ELISA kit (Shanghai, China). Cat.No: E0042Hu. This kit uses an enzyme-linked immunosorbent assay (ELISA) based on biotin double-antibody sandwich technology to assay Human Interleukin 35 (IL-35). Interleukin-35(IL-35) was added to the wells, which were pre-coated with Interleukin 35 (IL-35), monoclonal antibody, and then incubated. After that, add anti-IL-35 antibodies labeled with biotin were added to unite with streptavidin-HRP, which forms an immune complex. Removal of unbound enzymes after incubation and washing. Upon adding substrates A and B, the solution turned blue and changed to yellow because of the effect of the acid. The shades of solution and the concentration of Human Interleukin 35 (IL-35) are positively correlated.

#### IL-39

IL-39 levels in GCF were measured using an IL-39 ELISA kit provided by the Bioassay Technology Laboratory, China (Cat. No E7444Hu. This kit is an Enzyme-Linked Immunosorbent Assay (ELISA). The plate was pre-coated with Human IL-39 antibody. The IL-39 present in the sample was added and bound to the antibodies coated on the wells. Biotinylated Human IL-39 Antibody was then added and bound to the IL-39 in the sample. Streptavidin-HRP was then added and bound to the Biotinylated IL-39 antibody. After incubation, unbound streptavidin-HRP was washed away during a washing step. The substrate solution was then added and the color developed in proportion to the amount of Human IL-39. The reaction was terminated by the addition of an acidic stop solution and the absorbance was measured at 450 nm.

### Sample size calculation

This power analysis used IL-35 levels at baseline and after 1 week as the primary outcome. Based on the results of Goswamy et al. (2022) [31] the mean and standard deviation were 0.563 (0.312) and 0.812 (0.358) ng/µL, respectively. The resulting effect size (dz) is 0.738. Using an alpha (α) level of (5%) a power of 80%, the minimum estimated sample size was 17 subjects per group using an alpha (α) level of 5%. The sample size was increased to 19 subjects per group to compensate for a dropout rate of 10% after 1 month. Sample size calculation was performed using G*Power Version 3.1.9.2.

### Statistical analysis

Numerical data were explored for normality by checking the distribution of data and using tests of normality (Kolmogorov–Smirnov and Shapiro–Wilk tests). All data showed a normal (parametric) distribution. Data are presented as the mean ± standard deviation (SD). One-way ANOVA, Student’s *t*-test, and repeated measures ANOVA were used to compare between the groups as well as to study the changes after treatment within each group. Bonferroni’s post hoc test was used for pairwise comparisons when ANOVA was significant. Pearson’s correlation coefficient was used to determine the correlation between clinical parameters and interleukin levels. Qualitative data are presented as frequencies and percentages. The chi-square test was used to compare the groups. A Receiver Operating Characteristic (ROC) curve was constructed to determine the cutoff value for the two Interleukins to differentiate between Periodontitis and DM, Periodontitis, and the control group. ROC curve analysis was performed using the MedCalc® Statistical Software version 19.5.1 (MedCalc Software Ltd., Ostend, Belgium; https://www.medcalc.org; 2020). The significance level was set at *P* ≤ 0.05. Statistical analysis was performed using IBM SPSS Statistics for Windows, Version 23.0. Armonk, NY: IBM Corp.

## Results

### Base line characteristics

There was a statistically significant difference between the mean ages of the three groups. Pairwise comparisons between the groups revealed that there was no statistically significant difference between Periodontitis and DM and Periodontitis groups; both groups showed statistically significantly higher mean age values than the control group. There were no statistically significant differences between the sex distributions of the three groups. There was no statistically significant difference in the PD, CAL, BOP, and PI values between the Periodontitis and DM and Periodontitis groups. Both groups showed statistically significantly higher mean clinical parameter values than control group whether before or after treatment (Table 1). In the Periodontitis and DM groups, the mean (SD) HbA_1_c level was 8.78 (1.57%). The mean (SD) values for fasting blood glucose level were 178.95 (53.9) mg/dL.

**Table 1:** Demographic data and clinical parameters of included groups

	Periodontitis and DM (*n* = 19)	Periodontitis (*n* = 19)	Control (*n* = 19)	*P*-value
Age (years)				
Mean (SD)	48.2 (8.1)^A^	46.2 (9.2)^A^	32.5 (3.2)^B^	< 0.001*
Gender [n (%)]				
Male	5 (26.3%)	9 (47.4%)	9 (47.4%)	0.312
Female	14 (73.7%)	10 (52.6%)	10 (52.6%)	
PD (mm) [Mean (SD)]				
Before treatment	5.91 (0.49)^A^	5.91 (0.34)^A^	1.47 (0.51)^B^	< 0.001*
After treatment	3.87 (0.38)^A^	3.67 (0.35)^A^		< 0.001*
CAL (mm) [Mean (SD)]				
Before treatment	6.58 (0.78)^A^	6.57 (0.57)^A^	0 (0)^B^	< 0.001*
After treatment	3.65 (0.53)^A^	3.47 (0.37)^A^		< 0.001*
BOP (%) [Mean (SD)]				
Before treatment	90.47 (8.68)^A^	90.46 (4.02)^A^	5.9 (1.2)^B^	< 0.001*
After treatment	16.6 (1.3)^A^	15.9 (1.5)^A^		< 0.001*
PI (%) [Mean (SD)]				
Before treatment	89.16 (5.83)^A^	86.56 (4.4)^A^	5.6 (1.3)^B^	< 0.001*
After treatment	14.8 (1.1)^A^	14.2 (1.3)^A^		< 0.001*

*Significant at *P* ≤ 0.05; different superscripts in the same row indicate statistically significant differences between groups

### Interleukin levels

#### As regards IL-35 levels whether before or after treatment

There was a statistically significant difference between the mean IL-35 levels in the three groups (*P*-value < 0.001, effect size = 0.932) and (*P*-value < 0.001, effect size = 0.813), respectively. Pairwise comparisons between groups revealed that the control group had the highest mean IL-35 levels. The periodontitis group showed a significantly lower mean level. The periodontitis and DM groups showed the lowest mean IL-35 levels. Regarding the change in IL-35 levels in periodontitis and DM, and periodontitis groups, there was a statistically significant increase in IL-35 levels after treatment (*P*-value < 0.001, effect size = 0.787) and (*P*-value < 0.001, effect size = 0.891), respectively. The periodontitis and DM groups showed a significantly lower mean increase in IL-35 levels than that in the periodontitis group (*P*-value = 0.001, effect size = 1.287) (Table 2).

**Table 2:** Descriptive statistics comparing levels of both interleukins in included groups

Interleukin	Time	Periodontitis and DM (*n* = 19)		Periodontitis (*n* = 19)		Control (*n* = 19)		*P*-value	Effect size
		Mean	SD	Mean	SD	Mean	SD		
IL-35	Before treatment	18.9^C^	3.4	26.4^B^	5.3	67.4^A^	8.1	< 0.001*	Partial Eta Squared = 0.932
	After treatment	31.5^C^	6	45.1^B^	7.7	67.4^A^	8.1	< 0.001*	Partial Eta Squared = 0.813
	Change	12.6	4.7	18.7	4.9	-	-	0.001*	d = 1.287
*P*-value (Effect of time)		< 0.001*		< 0.001*					
Effect size (Partial Eta Squared)		0.787		0.891					
IL-39	Before treatment	171.7^A^	30.4	128^B^	19.7	24.3^C^	0.7	< 0.001*	Partial Eta Squared = 0.902
	After treatment	137.3^A^	28.3	100.1^B^	16.7	24.3^C^	0.7	< 0.001*	Partial Eta Squared = 0.866
	Change	− 34.5	8.7	− 27.9	9.6	-	-	0.064	d = 0.650
*P*-value (Effect of time)		< 0.001*		< 0.001*					
Effect size (Partial Eta Squared)		0.881		0.829					

*Significant at *P* ≤ 0.05; different superscripts in the same row indicate statistically significant differences between groups

#### Concerning IL-39 levels whether before or after treatment

There was a statistically significant difference between the mean IL-39 levels in the three groups (*P*-value < 0.001, effect size = 0.902) and (*P*-value < 0.001, effect size = 0.866), respectively. Pairwise comparisons between the groups revealed that the Periodontitis and DM groups had the highest mean IL-39 levels. The Periodontitis group showed a significantly lower mean level. The control group had the lowest mean IL-39 levels. Regarding the change in IL-39 levels in periodontitis and DM, and periodontitis groups, there was a statistically significant decrease in IL-39 levels after treatment (*P*-value < 0.001, effect size = 0.881) and (*P*-value < 0.001, effect size = 0.829), respectively. There was no statistically significant difference between the mean decrease in IL-39 levels in Periodontitis and DM as well as in the periodontitis group (*P*-value = 0.064, effect size = 0.650) (Table 2).

### Diagnostic accuracy of the two Interleukins to differentiate between periodontitis and DM and control groups

**Fig. 1:**
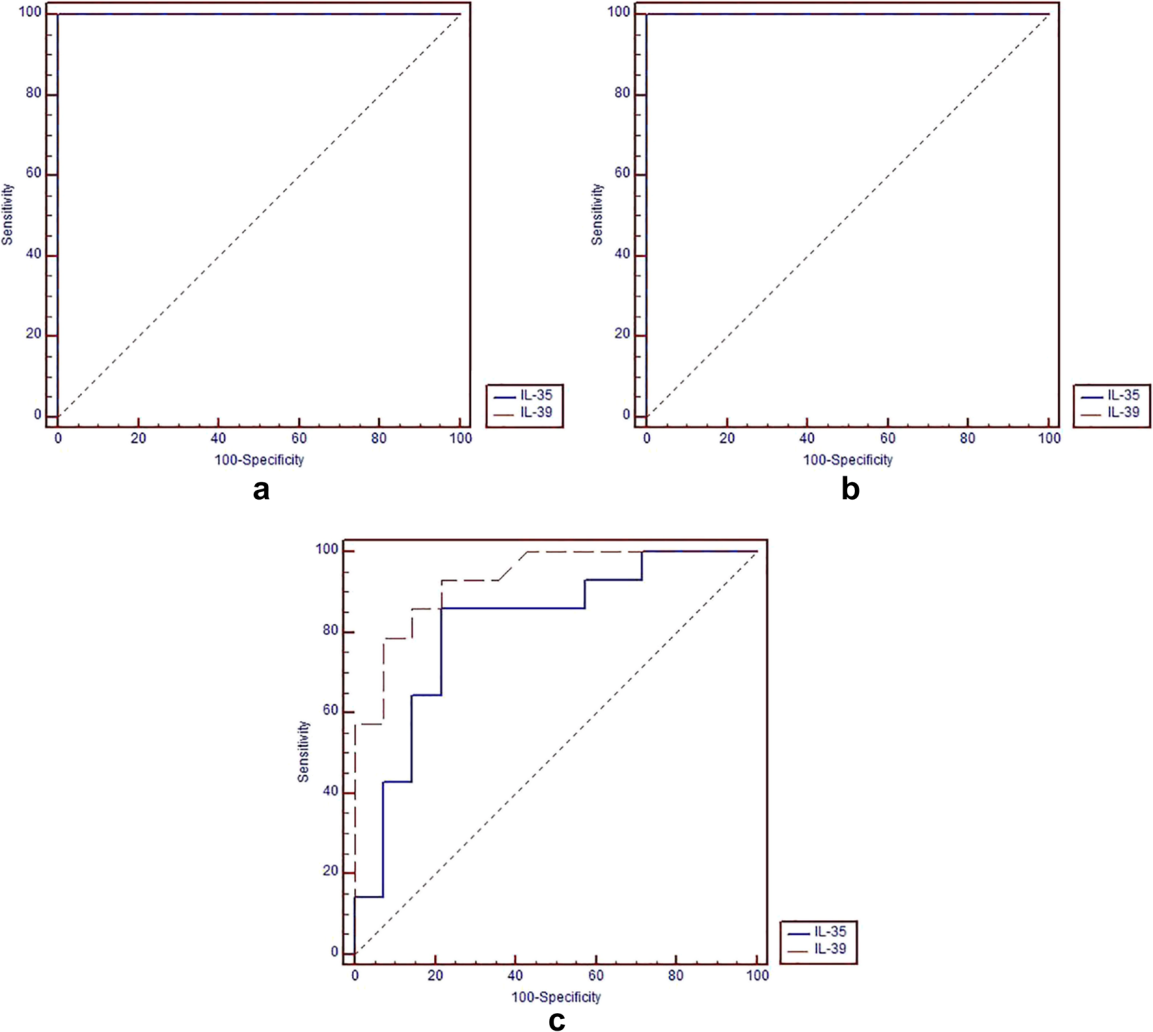
**a** ROC curve of the two interleukins for differentiation between Periodontitis and DM and control groups, **b** ROC curve of the two interleukins for differentiation between Periodontitis and control groups, **c** ROC curve of the two interleukins for differentiation between Periodontitis and DM and Periodontitis groups

The ROC curve analysis of the two Interleukins for differentiation between Periodontitis and DM and the control group is presented in Table 3 and Fig. 1(a). ROC curve analysis showed that both interleukins had a diagnostic accuracy of (100%).

**Table 3:** ROC curve analysis differentiating periodontitis and DM group from healthy control

Interleukin	Cut-off value	Sensitivity %	Specificity %	+ PV %	− PV %	Diagnostic accuracy %	AUC	95% CI
IL-35	≤ 27.08	100	100	100	100	100	1	0.858–1
IL-39	> 24.92	100	100	100	100	100	1	0.858–1

+ *PV*, positive predictive value; − *PV*, negative predictive value

### Diagnostic accuracy of the two interleukins to differentiate between periodontitis and control groups

The ROC curve analysis of the two Interleukins for differentiation between the periodontitis and control groups is shown in Table 4 and Fig. 1(b). ROC curve analysis showed that both interleukins had a diagnostic accuracy of (100%).

**Table 4:** ROC curve analysis discriminating periodontitis group from healthy control

Interleukin	Cut-off value	Sensitivity %	Specificity %	+ PV %	− PV %	Diagnostic accuracy %	AUC	95% CI
IL-35	≤ 35.4	100	100	100	100	100	1	0.858–1
IL-39	> 24.92	100	100	100	100	100	1	0.858–1

### Diagnostic accuracy of the two interleukins to differentiate between periodontitis and DM and periodontitis groups

The ROC curve analysis of the two interleukins for differentiation between Periodontitis and DM and Periodontitis groups is presented in Table 5 and Fig. 1(c). ROC curve analysis showed that IL-35 had lower diagnostic accuracy (82.2%) than IL-39 (85.8%). However, there was no statistically significant difference between the two interleukins in the differentiation between Periodontitis and DM and Periodontitis groups (*P*-value = 0.189).

**Table 5:** ROC curve analysis differentiating both periodontitis groups

Interleukin	Cut-off value	Sensitivity %	Specificity %	+ PV %	− PV %	Diagnostic accuracy %	AUC	95% CI
IL-35	≤ 22.45	85.7	78.6	80	84.6	82.2	0.811	0.619–0.933
IL-39	> 138.8	92.9	78.6	81.3	91.7	85.8	0.931	0.768–0.992

+ *PV*, positive predictive value; − *PV*, negative predictive value

### Correlation between clinical parameters and interleukin levels

Regarding IL-35, there was a statistically significant inverse (negative) correlation between PD, CAL, BOP, PI, and IL-35 levels before and after treatment. While for IL-39; there was a statistically significant direct (positive) correlation between PD, CAL, BOP, PI, and IL-39 levels before and after treatment (Table 6).

**Table 6:** Results of Pearson’s correlation coefficient for the correlation between clinical parameters and interleukin levels

Time	Clinical parameters	IL-35		IL-39	
		Correlation coefficient (*r*)	*P*-value	Correlation coefficient (*r*)	*P*-value
Before treatment	PD	− 0.925	< 0.001*	0.886	< 0.001*
	CAL	− 0.936	< 0.001*	0.892	< 0.001*
	BOP	− 0.948	< 0.001*	0.917	< 0.001*
	PI	− 0.953	< 0.001*	0.914	< 0.001*
After treatment	PD	− 0.804	< 0.001*	0.856	< 0.001*
	CAL	− 0.820	< 0.001*	0.871	< 0.001*
	BOP	− 0.835	< 0.001*	0.861	< 0.001*
	PI	− 0.822	< 0.001*	0.883	< 0.001*

*Significant at* P* ≤ 0.05

## Discussion

Periodontitis is a chronic inflammatory disease, in which the locally implemented host immune response against periodontopathic bacteria plays an essential role in the onset and progression of the disease via the release of an array of immune mediators. Among these mediators are the proinflammatory and anti-inflammatory cytokines. Currently, host modulation therapy as an anti-cytokine therapy has proven its capability to re-establish the balance between various mediators, thus restoring normal microflora [32]. Consequently, it opens the door for the research field to seek advanced methods that can diagnose, monitor, and predict the response to treatment in addition to exploring new management modalities. IL-35, as well as the most recently discovered cytokine, IL-39, have drawn considerable attention in recent years.

GCF samples as non-invasive method of obtaining samples could aid in differentiation of active sites, forecast potential tissue destruction as well as diagnose early signs of periodontitis. Thus, it may have a prospective role in the development of new therapeutic modalities through host modulatory drugs for periodontal disease treatment that aid in presenting a more individualized targeted treatment for oral health [21].

IL-35, a novel immunoregulatory cytokine with anti-inflammatory capacities, had been detected in periodontitis patients and a possible role in maintaining the homoeostasis of the local immune microenvironment in periodontal tissues had been expected [33, 34]. Moreover, preclinical studies had reported anti-inflammatory criteria for it like inhibiting the production of two essential proinflammatory interleukin, IL-6 and IL-8 [35]. As well as an animal study was performed by Cafferata et al. 2020 showed that IL-35 inhibited alveolar bone resorption via modulation of the periodontal Th17/Treg imbalance [36]. The most recent systematic review in 2021 verified the obvious role of IL-35 in pathobiology of periodontitis although no specific mechanism was established [37]. The newly discovered member of IL-12 family, IL-39, has been advocated as proinflammatory marker in humans [11]. Regarding periodontal diseases, only one study has estimated its GCF levels in periodontal diseases and health [5]. Accordingly, the goal of our study was assessment of GCF level of both biomarkers in healthy and diseased groups and to follow up their levels postoperatively.

In the present study, the level of IL-35 was consistent with previous studies, where Koseoglu et al., 2015 found that the concentration of IL-35 in the GCF was higher in the healthy group than in the periodontitis group. They assumed that increased levels of IL-35 could play an important role in preventing periodontal inflammation and maintaining periodontal health [38]. Another study by Kaustubh et al., 2017 revealed a higher GCF level of IL-35 in the healthy group than in the gingivitis group [39]. A study by the same authors evaluated and compared the levels of IL-35 in GCF in patients with chronic gingivitis and chronic periodontitis. The GCF IL-35 levels were significantly higher in the chronic gingivitis group than in the chronic periodontitis group; therefore, they concluded that the levels of IL-35 decrease with an increase in the inflammatory status; thus, it might play a role in controlling gingival inflammation and maintaining periodontal health [40]. We supposed that the high level of IL-35 in healthy sulcus confirms its anti-inflammatory role via interaction with the host mechanisms to maintain periodontal health; however, its low level in the periodontitis group is due to the active process of inflammatory reactions.

Similarly, a recent study by Kaustubh et al., 2022 was carried out to evaluate and compare the GCF levels of IL-35 in periodontally healthy subjects, patients with gingivitis, and chronic periodontitis, and to assess IL-35 as a marker for the identification of periodontal disease activity. The authors observed that IL-35 levels were significantly higher in healthy subjects than in gingivitis and chronic periodontitis groups and that IL-35 levels were negatively correlated with inflammatory status, which supports our hypothesis that IL-35 is an anti-inflammatory biomarker in the control of disease activity [41].

An earlier study reported conflicting results regarding IL-35 levels and compared the IL-35 levels in gingival tissues of healthy controls and patients with chronic and aggressive periodontitis, and found that IL-35 was expressed in all samples with the highest level in chronic periodontitis [42]. The difference in our results may be attributed to dissimilarities in the study design, in addition to the difference in sample nature.

Another controversial result was demonstrated in an investigation conducted by Mitani et al., 2015 where IL-35 was significantly higher in GCF from patients with periodontitis than in healthy participants. They suggested that IL-35 levels in GCF reflect broad biological responses that occur during periodontitis, such as short periods of activity and long periods of quiescence; therefore, the GCF sample might be collected from quiescent sites as tissue repair takes place. Their explanation also supports our results and the assumption of the anti-inflammatory role of the estimated biomarker. It is believed that increased IL-35 levels may be necessary to resolve inflammation in periodontitis [43].

Regarding periodontitis and DM subjects, IL-35 levels throughout our study showed the lowest level, and postoperatively revealed the lowest significant increase, which returned to the fact that type 2 diabetes is associated with a high Th-2/Th-1 ratio and adversely influences the local expression of molecules involved in anti-inflammatory and healing processes [44]. One previous study in literature had assessed the serum level of IL-35 in patients with periodontal disease, including type 2 diabetic patients with a control group. Inconsistent results to our data were reported, as no relationship between both diseases and level of IL-35 was proved [45]. We supposed that it could be due to different evaluated body fluids.

Concerning the interventional studies, a recent study conducted by Goswamy et al., 2022 registered an increase in concentration of IL-35 postoperatively. These results confirm the increase in IL-35 levels within the gingival sulcus, as the resolution of inflammation occurred following scaling and root planing, thus confirming the anti-inflammatory function of the examined cytokines [31].

In contrast to our findings, Raj et al. 2018 had assessed the IL‑35 levels in GCF of healthy controls and gingivitis and chronic periodontitis subjects, and their results revealed that IL‑35 concentration in GCF among chronic periodontitis subjects was the highest, followed by gingivitis and healthy subjects; moreover, the authors established a positive correlation between clinical parameters and GCF IL-35 levels as patients receiving scaling and root planing showed a significant reduction in IL‑35 levels as compared to pre-operative results in chronic periodontitis subjects. They speculated that the regression of inflammation resulted from a decline in the expression of Treg cells, which were recruited for their role in arresting destruction, leading to diminished IL-35 expression [2]. This study and ours have a few variations, a notable one being the inclusion criteria that were not determined in their study, which may be the reason for our contradictory results. In the same year, Ustun et al. showed a significant reduction in the GCF level of IL-35 after scaling and root planning in patients with chronic periodontitis; however, their methodology was not in accordance with our research procedures [46].

Similarly, inconsistent results were presented by Varghese et al., 2021 as they assessed the GCF levels of IL-35 in healthy subjects and patients with chronic periodontitis, and compared the levels of IL-35 before and after scaling and root planing. They reported higher levels of biomarkers in chronic periodontitis patients than in healthy individuals and a significant reduction after scaling and root planing. They believed that the increased level of IL-35 in the chronic periodontitis group was due to its increased expression by activated T cells following inflammation. After induction, the suppressive action of IL-35 on inflammatory conditions is stimulated. Postoperatively, a reduction in inflammatory responses may occur; therefore, the induction of IL-35 by T cells in peripheral tissues can also be restricted^.^ [47]. The diverse results may be due to the time of post operative sample collection, as we collected it after 4 weeks, which may be the time interval of increased expression referred to by the authors of the aforementioned study.

In agreement with our results, IL-39 levels were the highest in the periodontitis and DM groups, and the periodontitis group showed a significantly lower mean level, followed by the control group, which showed the lowest level with significant differences. In agreement with our results, a study performed by Sari et al., 2022 was aiming to evaluate the GCF IL-39 levels in periodontal diseases and health and to correlate them with the GCF levels of IL-1β and periostin. Their findings showed that IL-39 levels were higher in the periodontitis and gingivitis groups than in the periodontal health group, suggesting that IL-39 may play a role in the periodontal inflammation process, consequently confirming our expectations regarding the proinflammatory nature of IL-39 [5].

Many previous studies have confirmed our point of view, as their results pointed to the pro inflammatory role of this biomarker; for instance, increased serum levels of IL-39 in acute coronary syndrome patients than in healthy subjects, and a positive correlation with hs-CRP was reported by Luo et al., 2017 [10]. Furthermore, patients with relapsing–remitting multiple sclerosis and neuromyelitis optica spectrum disorders revealed higher serum levels of IL-39 than the healthy group [48].

Furthermore, a recent study reported that IL-39 levels were elevated in the serum of T2DM patients, suggesting that IL-39 can be considered a novel predictor of T2DM and/or a therapeutic target in the disease, which supports our significant data concerning periodontitis and DM groups [49]. No previous interventional periodontal studies have estimated the level of IL-39; therefore, our data could be considered as primary records in this context. According to our obtained pre-and post-operative results, we thought that IL-39 may have a therapeutic target potential in periodontal diseases, signified by its decreased level postoperatively. A previous in vitro study observed that anti-IL-39 antibodies have a curative role in lupus-like disease in mice, which supports our hypothesis [50]. The insignificant difference observed between postoperative results of IL-39 between both groups of periodontitis requires further studies to illuminate it.

Non-surgical periodontal therapy is a critical aspect of periodontal disease management, targeted at the removal of the etiologic factor, thereby arresting disease progression and the re-establishment of biologically acceptable root surfaces for healing [51]. As one of the treatment modalities for periodontitis with or without diabetes, significant improvement in clinical parameters can be seen in non-diabetic as well as diabetic patients following NSPT [52]. In our study, non-surgical periodontal therapy appeared to substantially augment the levels of IL-35 and downregulate the levels of IL-39 with significant values within the gingival crevice, which might be due to the healing of the periodontal tissues, although not reaching control levels. Our explanation for this result is that NSPT does not regenerate the normal periodontium and halts disease, so a considerable significant difference is observed even though it does not reach the healthy control level.

In the ROC analysis, both IL-35 and IL-39 showed meaningful diagnostic accuracy between the control and disease groups; thus, their GCF levels could have a prospective position in diagnosis. Additionally, these two biomarkers can distinguish between the included disease groups with high diagnostic accuracy.

The short time interval, relatively small sample size (including only stage III participants), and the absence of periodontally healthy diabetic participants may be limitations of the present study.

Based on our observations, IL-35 could be considered as a valuable anti-inflammatory biomarker that aids in the arrest of periodontal destruction. Furthermore, our results corroborate the hypothesis that IL-39 is a proinflammatory marker involved in the pathogenesis and progression of periodontitis. Although more investigations with a longer duration and larger sample size are required to confirm our first trial to observe the GCF level of IL-39 postoperatively, future studies are recommended to clarify the exact mechanism by which both interleukins contribute to the pathogenesis of periodontal disease. In addition to estimating the levels of biomarkers in the blood samples, this may support our conclusions.

## Data Availability

Data used or analyzed in this investigation are included within the manuscript or available upon reasonable request from the corresponding author.
